# Attentional Modulation of Vision Versus Proprioception During Action

**DOI:** 10.1093/cercor/bhz192

**Published:** 2019-10-16

**Authors:** Jakub Limanowski, Karl Friston

**Affiliations:** Wellcome Centre for Human Neuroimaging, Institute of Neurology, University College London, London, UK

**Keywords:** action, attention, dynamic causal modeling, sensorimotor integration

## Abstract

To control our actions efficiently, our brain represents our body based on a combination of visual and proprioceptive cues, weighted according to how (un)reliable—how precise—each respective modality is in a given context. However, perceptual experiments in other modalities suggest that the weights assigned to sensory cues are also modulated “top-down” by attention. Here, we asked whether during action, attention can likewise modulate the weights (i.e., precision) assigned to visual versus proprioceptive information about body position. Participants controlled a virtual hand (VH) via a data glove, matching either the VH or their (unseen) real hand (RH) movements to a target, and thus adopting a ``visual'' or ``proprioceptive'' attentional set, under varying levels of visuo-proprioceptive congruence and visibility. Functional magnetic resonance imaging (fMRI) revealed increased activation of the multisensory superior parietal lobe (SPL) during the VH task and increased activation of the secondary somatosensory cortex (S2) during the RH task. Dynamic causal modeling (DCM) showed that these activity changes were the result of selective, diametrical gain modulations in the primary visual cortex (V1) and the S2. These results suggest that endogenous attention can balance the gain of visual versus proprioceptive brain areas, thus contextualizing their influence on multisensory areas representing the body for action.

## Introduction

To control our actions efficiently, our brain constructs a multisensory body representation based mainly on a combination of visual and proprioceptive cues (Ghahramani et al. 1997; Graziano and Botvinick 2002; Holmes and Spence 2004; Makin et al. 2008; Blanke et al. 2015). The underlying integration process can be well described in terms of flexible Bayesian inference (cf. Ernst and Banks 2002; Körding and Wolpert 2004), where individual sensory cues are weighted according to their relative precision (i.e., inversely proportional to the level of noise present in the signal; thus a model of sensory noise or reliability is an important part of internal models for sensorimotor control, Körding and Wolpert 2004; Ma et al. 2006; Bestmann et al. 2008). When reaching or grasping, for instance, we usually rely heavily on where and in which position we see our hand to be, because estimates of visual position are more precise—less noisy—than the proprioceptive modality (Sober and Sabes 2005; cf. van Beers et al. 1999). On the neuronal level, this integration process is thought to be implemented by the posterior parietal cortex (PPC) and its communication with visual and somatosensory brain areas (Wolpert et al. 1998; Graziano et al. 2000; Ehrsson et al. 2004; Beauchamp et al. 2010; Wasaka and Kakigi 2012; Gentile et al. 2013; Limanowski and Blankenburg 2015a, 2016, 2017).

However, we can sometimes choose which of our senses to focus on in a particular context—where to allocate our resources. This has been studied as “crossmodal” or “intersensory” attention (cf. Driver and Spence 2000; Rowe et al. 2002; Macaluso and Driver 2005; Talsma et al. 2010; Tang et al. 2016). Studies using perceptual paradigms have shown modulations of brain responses in early sensory levels when participants were instructed to attend to, for example, auditory, visual, or tactile features of a multisensory stimulus (Alho et al. 1994; Hötting et al. 2003; Foxe and Simpson 2005; Johnson and Zatorre 2005); sometimes supplemented by an inhibition of the processing of the currently task-irrelevant modality (Kawashima et al. 1995; Gaspelin and Luck 2018; cf. van Kemenade et al. 2016). Such effects appear to be mediated through selective changes of neuronal gain (i.e., of input-output balance) in sensory areas based on top-down signals from hierarchically higher areas, which are therefore usually interpreted as signatures of attention (Fries et al. 2001; Martinez-Trujillo and Treue 2004; Thiele and Bellgrove 2018). This sits well within predictive coding accounts of functional architectures, where attention is the mechanism by which the relative influence of prediction errors from various sensory modalities on updating internal models (e.g., a body representation) can be attenuated or enhanced by regulating the post-synaptic gain of superficial pyramidal cell populations encoding these errors (Friston and Kiebel 2009; Feldman and Friston 2010; Bastos et al. 2012; Auksztulewicz et al. 2017). Based on the above, one could speculate that the selection or weighting of visual and proprioceptive cues during action do not only depend on sensory precision, but may also be modulated “top-down” by attention. Tentative support for this notion comes from suggestions that the resolution of visuo-proprioceptive conflict may be accompanied by a temporary attenuation of responses in the somatosensory cortex (Bernier et al. 2009; Limanowski and Blankenburg 2015b; Zeller et al. 2016).

**Figure 1:**
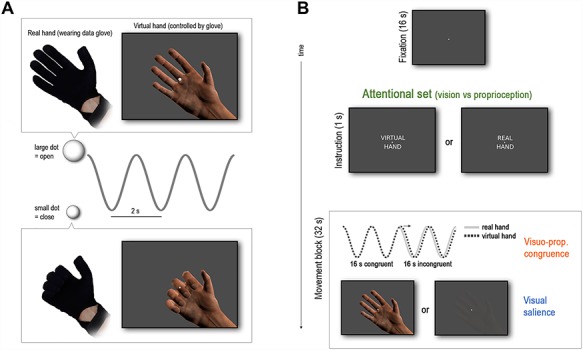
Experimental task and design. (*A*) Participants controlled a photorealistic right virtual hand (VH) model via an MR-compatible data glove, worn on their right real hand (RH, placed outside their view). Their task was to perform repetitive grasping movements paced by the oscillatory size change of a central fixation dot (continuous, sinusoidal shrinking-and-growing with 0.5 Hz frequency), i.e., close the hand when the dot shrunk and open it when it grew. (*B*) Trial structure and factors of the experimental design. Movements were performed in blocks of 32 s duration (16 movements), interspersed by 16 s fixation-only periods (including a brief presentation of the task instruction at the end). Prior to each movement block, participants were randomly instructed to perform the task with one of two goals in mind: matching the VH movements or the RH movements to the size change of the fixation dot (factor “attentional set”). Half-way throughout each movement block, an incongruence between the VH movements and the movements performed by the participant was introduced (i.e., a lag of the VH movements of 267 ms, indicated by the arrow; factor “visuo-proprioceptive congruence”); the resulting intersensory conflict increased task difficulty and attentional requirements. Furthermore, in half of the movement blocks, the visibility of the VH was reduced to manipulate the salience or precision of visual information (factor “visual salience”).

In the present study, we used a virtual reality environment together with functional magnetic resonance imaging (fMRI) recordings of brain responses and computational modeling to test whether an endogenous “attentional set” (cf. Posner et al. 1978; Corbetta and Shulman 2002; Foxe and Simpson 2005) can change the weights assigned to visual versus proprioceptive information about hand position during action. Participants moved a virtual hand (VH) with their unseen real hand (RH), performing repetitive grasping movements that were paced by a pulsating target (fixation dot, Fig. 1*A*). Participants were asked to match either the VH movements or their RH movements to the target’s oscillatory phase (factor “attentional set”). We moreover manipulated visuo-proprioceptive congruence (by introducing a lag into the VH movements, factor “visuo-proprioceptive congruence”) and visual salience (i.e., sensory precision, by varying the visibility of the VH, factor “visual salience”, Fig. 1*B*). Our main hypothesis (H1a) was that—depending on the task—participants would adopt an attentional set to prioritize either visual or proprioceptive information (i.e., in the VH task, visual information should be accumulated by bodily representations, whereas it should be “ignored” in the RH task). In terms of brain activity, we hypothesized (H1b) that attentional set would be reflected by differential activity in visual versus proprioceptive areas; and that the PPC would integrate visual movement information into the body representation—reflected by increased responses in the VH task—especially during periods of visuo-proprioceptive incongruence. Furthermore, we hypothesized (H1c) that this effect would interact with the other experimental factors, that is, with visuo-proprioceptive conflict (participants needed to resolve this conflict only in the VH task) and with visual salience (precise visual stimuli should make the VH task easier and the RH task more difficult). Finally, we hypothesized (H2) that responses in sensory areas would reflect selective changes in neuronal gain mediated by top-down “attentional” mechanisms.

## Materials and Methods

### Participants

A total of 17 healthy, right-handed volunteers (8 female, mean age = 27 years, range = 21–37, all with normal or corrected-to-normal vision) participated in the experiment, after providing written informed consent; 1 participant aborted the experiment due to feeling unwell and was excluded from data analysis, resulting in a final sample size of 16. The sample size was adopted from a recent related fMRI experiment using a similar virtual reality-based grasping task (Limanowski et al. 2017), in which we detected significant (*P* < 0.05, corrected for multiple comparisons) activations in the visual and multisensory areas that were of primary interest here. The experiment was approved by the local research ethics committee (University College London) and conducted in accordance with this approval.

### Experimental Setup and Procedure

During the experiment, participants lay inside the scanner wearing an magnetic resonance-compatible data glove (5DT Data Glove MRI, 1 sensor per finger, 8 bit flexure resolution per sensor, 60 Hz sampling rate, communication with the computer via Universal Serial Bus with approx. 10 ms delay) on their right hand (placed in a comfortable position across the chest, outside of the participants’ field of view [FOV]). The data glove measured the participant’s individual finger flexions via sewn-in optical fiber cables; i.e., light was passed through the fiber cables and to one sensor per finger—the amount of light received varied with finger flexion. Prior to scanning, the glove was carefully calibrated to fit each participant’s movement range (if necessary this was repeated between runs). The glove data was fed to a photorealistic virtual right hand model (cf. Limanowski et al. 2017), which was thus moveable by the participant in real-time. The VH, a fixation dot, and the task instructions were presented via a projector on a screen visible to the participant via a mirror attached to the head coil (1280 × 1024 pixels resolution, screen distance 62 cm, image size approx. 24 × 18°, approx. 40 ms projector latency). The virtual reality task environment was instantiated in the open-source 3D computer graphics software Blender (http://www.blender.org) using its Python programming interface. An eye tracker (EyeLink, SR Research) was used to monitor the participants’ eye position online, to ensure they maintained central fixation and did not close their eyes. The participants’ RH movements were further monitored by a separate video camera to ensure hand position was not changed between runs. The VH movements were also mirrored to a computer screen outside the scanner room—thus the VH and RH movements were always monitored by the experimenter. The participants were able to adhere to the dot’s pulsation frequency within reasonable limits (Supplementary Fig. 3).

The participants’ task was to perform repetitive right-hand grasping movements paced by the oscillatory size change of a central fixation dot; this was effectively a non-spatial phase matching task (Fig. 1*A*). The fixation dot continually decreased-and-increased in size sinusoidally (12% size change) with 0.5 Hz frequency. The participants had to follow the size changes with right-hand grasping movements; i.e., to close the hand when the dot shrunk and to open the hand when the dot grew. We chose the fixation dot as the target to ensure that participants had to look at the center of the screen—thus, also at the VH—under both instructions; the dot’s phasic change in size was chosen as a more abstract, non-spatial target quantity than, for example, a diagonally moving target in previous studies (Limanowski et al. 2017).

The task was performed in blocks of 32 s (16 movements; the last movement was signaled by a brief blinking of the fixation dot), separated by 16 s rest periods, during which only the fixation dot (static) was visible. Half-way throughout each movement block, a visuo-proprioceptive incongruence was introduced between the participant’s movements and the movements of the VH; during the first half of each movement block (first eight movements), the VH moved according to the participant’s hand movements, whereas during the second half (last eight movements), the VH’s movements were delayed with respect to the actual movement by adding a 267 ms lag. This value was inherited from a recent study using a similar virtual reality task (Limanowski et al. 2017), which showed that participants reliably recognized the VH and RH movements as incongruent when applying this amount of lag. Here, we likewise ensured that all participants were aware of the incongruence before scanning. The visuo-proprioceptive incongruence was gradually increased over 1 s between the eighth and ninth movement to avoid sudden changes in the VH position. The transition from congruent to incongruent visuo-proprioceptive mapping during movement was adopted from our previous study (Limanowski et al. 2017). Thus, participants always started with the “easier” condition and transitioned to the “harder” condition (cf. Supplementary Fig. 2 for ratings of task difficulty). As all movement blocks had the same, predictable structure, we expected that participants would adopt a specific attentional set—depending on the instructed modality—preceding each movement block, and maintain it throughout. We did not introduce transitions from incongruent to congruent mapping for two reasons: firstly, to preclude behavioral after-effects of visuo-motor adaptation (cf. Ingram et al. 2000) that would carry over to the congruent blocks in the VH task (but not in the RH task). Secondly, we were worried that participants could relax their attentional focus for the “easier” congruent period after having completed the incongruent condition. However, please note that due to introducing visuo-proprioceptive congruence in this fixed order, any related main effects and interactions had to be interpreted with some caution, and are therefore presented as supplementary results only.

Crucially, participants had to perform the phase matching task with one of two goals in mind: to match the dot’s phase with the VH movements or with their unseen RH movements. Accordingly, the written task instructions (“VIRTUAL HAND”/“REAL HAND”) were presented 2 s before each respective movement block for 1 s (Fig. 1*B*). With these instructions, we aimed to induce a specific attentional set in our participants; i.e., a different weighting of visual versus proprioceptive movement cues. The VH task required a focus on the visual movement information from the VH, because vision of the hand was necessary for correct phase matching. In the RH task, conversely, visual hand information was task-irrelevant—effectively constituting a cross modal distractor during the incongruent movement periods—and therefore performance would benefit from ignoring the VH and focusing on the unseen RH movements. Note that the induced attentional set was particularly relevant for moving during periods of visuo-proprioceptive incongruence, because participants had to decide which of the two hands (VH or RH) to align with the phase of the fixation dot.

The final factor in our design involved lowering the visibility (alpha level) of the VH in half of the blocks. This was done to manipulate visual salience (i.e., sensory precision), following reported salience (i.e., contrast gain) effects in visual cortex (Gardner et al. 2005; Brown and Friston 2012). The “low visibility” setting was individually determined so that the participants reported that they were barely able to see the VH but still could perform the task. This resulted in a balanced 2 × 2 × 2 factorial design with the factors “attentional set” (VH vs. RH task), “visuo-proprioceptive congruence” (congruent vs. incongruent hand positions), and “visual salience” (high vs. low visibility). Each condition was presented three times per run—attentional set and visual salience were randomized, while visuo-proprioceptive congruence was a fixed-order, nested factor (see above)—which resulted in 10 min run length. Participants were trained extensively prior to scanning to ensure that they had understood the task instructions. Participants completed five runs in total resulting in 15 repetitions of each condition. After, scanning, participants were asked to indicate whether they found each task (VH and RH) easier to perform under high or low visibility (of the VH). The ratings were given on a seven-point visual analogue scale ranging from “Easier under low visibility” to “Easier under high visibility”.

### Behavioral Data Analysis

The participants’ ratings of visibility-dependent task difficulty (pooled across congruent and incongruent movement periods) were evaluated for statistically significant differences using a non-parametric Wilcoxon’s signed-rank test due to non-normal distribution of the data. We also tested for potential differences in movement amplitude across conditions: for each participant, we calculated the difference between maximum extension and maximum flexion for all movements in each condition; the resulting participant-specific condition averages were entered into a 3-way analysis of variance (ANOVA) with the factors attentional set, visuo-proprioceptive congruence, and visual salience. Furthermore, although our main concern was the manipulation of endogenous attentional focus rather than visuo-motor adaptation, we tested for differences in phase-matching performance during visuo-proprioceptive conflict between the VH and RH tasks. We calculated the average shifts between the phase of the VH movements and the phase of the fixation dot (cf. Limanowski et al. 2017), which were entered into an ANOVA with the factors attentional set and visual salience.

### fMRI Data Preprocessing and Analysis

The fMRI data were acquired using a 3T scanner (Magnetom TIM Trio, Siemens) equipped with a 64-channel head coil. T_2_^*^-weighted images were acquired using a gradient echo-planar imaging sequence (voxel size = 3 × 3 × 3 mm^3^, matrix size = 64 × 72, TR = 3.36 s, TE = 30 ms, flip angle = 90°). For each participant, we recorded 5 runs of 175 functional images, a field map (double-echo FLASH sequence, voxel size = 3 × 3 × 3 mm^3^, FOV = 192 × 192 mm^2^, 64 slices, TE_1_ = 10 ms, TE_2_ = 12.46 ms) and a T_1_-weighted structural image (3D magnetization-prepared rapid gradient-echo, voxel size = 1 × 1 × 1 mm^3^, FOV = 256 × 256 mm^2^, 176 slices, TR = 2530 ms, TE = 3.34 ms, flip angle = 7°). fMRI data were preprocessed and analyzed using SPM12.5 (www.fil.ion.ucl.ac.uk/spm/). Artifacts at the slice-level were corrected using the ArtRepair toolbox (Mazaika et al. 2009; on average 1.3% of slices were corrected). Images were corrected for slice acquisition time differences, realigned and unwarped, normalized to MNI space and resliced to 2 mm isotropic voxels, spatially smoothed with an 8 mm full width at half maximum Gaussian kernel, detrended (Macey et al. 2004) and images featuring excessive (0.5 mm scan-to-scan) movement were interpolated (ArtRepair; on average 2.3% of volumes were corrected).

We fitted a general linear model (GLM, 128 s high-pass filter) to each participant. The movements of each factor level of attentional set and visual salience were modeled with boxcar functions of 2 s duration each, resulting in a continuous block-like regressor for each 32 s movement period. The nested factor visuo-proprioceptive congruence was modeled via parametric modulators of each 32 s movement block: each movement regressor received a parametric modulator, which modeled the effect of incongruent > congruent hand positions (over and above that explained by the block regressor), i.e., −1 for the first half of each 32 s movement block and 1 for the second half. See Supplementary Figure 5 for an example of a first-level design matrix. The realignment parameters were added to the GLMs alongside a regressor modeling the instructions (as regressors of no interest). The realignment parameters were not systematically correlated with any of the experimental conditions (Supplementary Table 3). For each subject, we calculated contrast images of each block regressor and parametric modulator against baseline; these were entered into two equivalent group-level flexible factorial designs, each with the factors attentional set and visual salience and an additional factor modeling the subject constants. Thus, we were able to test for general effects of each movement block, and those specific to the incongruent versus congruent movement periods separately.

Activations in the whole brain obtained from group-level contrasts were assessed for statistical significance using a voxel-wise threshold of *P* < 0.05, family-wise error (FWE) corrected for multiple comparisons. Due to our strong prior hypothesis that an endogenous attentional focus on the RH would manifest itself as a modulation of proprioceptive gain (see introduction), we restricted our search space for effects of the RH > VH task to the somatosensory cortices contralateral to the moving hand; i.e., we looked for activation differences within an anatomical mask comprising areas BA 1, 2, 3b, and OP 1–4. To identify areas showing a main effect of visuo-proprioceptive congruence, we used a “null” conjunction contrast across all regressors coding for visuo-proprioceptive congruence in each condition. Finally, we looked for interaction effects between attentional set and the other experimental factors in a search space defined by all voxels showing a significant main effect of attentional set at *P* < 0.05, corrected for multiple comparisons. The resulting statistical maps were projected onto the mean normalized structural image or rendered on SPM12’s brain template. The unthresholded T-maps corresponding to the contrasts reported in the manuscript can be inspected online at https://neurovault.org/collections/4868/. The SPM Anatomy toolbox (Eickhoff et al. 2005) was used for anatomical reference.

### Dynamic Causal Modeling

Dynamic causal modeling (DCM) is a Bayesian framework that allows one to compare models of neuronal responses of a set of coupled brain regions that generate a prediction of measured brain activity; for example, predicted blood oxygenation level dependent (BOLD) signal time series. DCM thus allows one to test hypotheses about how observed changes in brain activity are caused by changes in effective connectivity within or among a set of brain regions under a specific network architecture.

**Figure 2:**
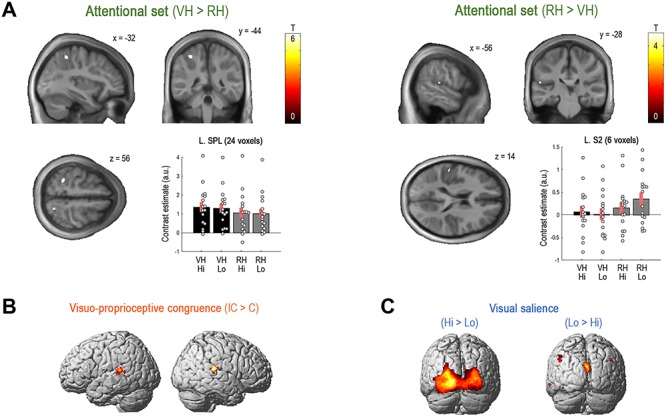
Significant brain activation differences. (*A*) Main effects of attentional set. During the VH > RH task, we observed significantly increased responses in the bilateral SPL; at uncorrected thresholds, further activation differences were observed in the left V5 and V1 (Supplementary Figs 5 and 6). Conversely, during the RH > VH task, there were significantly increased responses in the left S2. The plots show the contrast estimates for each condition with associated standard errors; the individual participants’ contrast estimates are shown as gray dots. VH/RH = virtual hand/real hand task; Lo/Hi = low/high visibility. (*B*) Visuo-proprioceptive incongruence > congruence increased responses in bilateral temporal regions and the left S2. (*C*) Visual salience (high > low) increased responses in primary and extrastriate visual regions. All SPMs and renders are thresholded at *P* < 0.05, FWE corrected for multiple comparisons. The corresponding unthresholded statistical maps can be viewed at https://neurovault.org/collections/4868/. See Table 1 for details.

We used DCM to test our hypothesis about changes in connectivity related to attentional set (H2). We focused our analysis on a left-lateralized network comprising visual, somatosensory, and multisensory areas identified by our statistical parametric mapping (SPM) results: the left primary visual cortex (V1), the left V5, the left secondary somatosensory cortex (S2), and the left superior parietal lobe (SPL). We included the left V1 and the left V5, in which effects of attentional set were significant at uncorrected thresholds (cf. Supplementary Figs 7 and 8). The effect size in the left V5 for the VH > RH contrast was qualitatively similar to estimates from our previous study (Limanowski et al. 2017; cf. Supplementary Figs 8 and 11). Moreover, both regions were significantly activated by visual input during the movement task per se (*P* < 0.05, corrected for multiple comparisons, cf. Supplementary Fig. 6). Likewise, both regions exhibited significantly increased responses during high-contrast visual stimuli (Fig. 2*C*); this effect was strongest in the V1, which we therefore included as the most plausible source for visual input in our model. Time series of activity in each region were summarized as the first eigenvariate of all voxels within a 4 mm radius sphere centered on the subject-specific local maximum within 10 mm Euclidean distance from the respective group maximum as identified in the SPM analysis. The individual peaks were identified by the following contrasts: VH > RH task for the V1, V5, and SPL; RH > VH task for the S2. For extraction of the time series, we concatenated the five runs of each participant (this yielded a single time series for each region, Supplementary Fig. 12). The time series were adjusted for effects of no interest; i.e., instruction periods, session means, and movement regressors.

For the DCM specification, we created separate first-level design matrices with a regressor coding for driving sensory inputs (common to all movement types), and regressors coding for modulatory inputs, i.e., for each of the experimental main effects and significant interactions of our design. For example, the main effect of attentional set was modeled by a regressor set to 1 for all VH movement blocks and − 1 for all RH movement blocks. The resulting parameter estimates can therefore be interpreted as relative differences in modulation, for example, a stronger modulation of a particular connection by the VH task than the RH task. See Supplementary Figure 13 for details.

**Figure 3:**
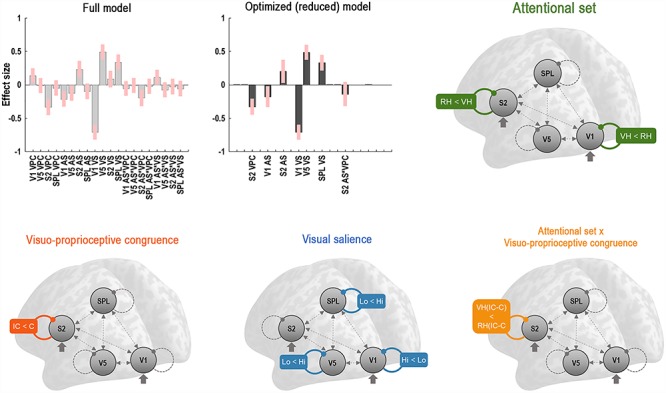
DCM results. Results of the BMR testing for all possible modulatory effects on regional self-connectivity. The plots show the parameter estimates with 90% posterior confidence intervals of the full model and the optimal reduced model (Bayesian model averages of retained parameters), indicating how strongly each coupling parameter was modulated by the respective main or interaction effect (AS = attentional set; VS = visual salience; VPC = visuoproprioceptive incongruence; see Supplementary Fig. 15 for details). The retained modulation effects are schematically depicted. Dashed lines indicate latent (endogenous) connectivity; thick arrows indicate driving inputs; colored lines indicate (nonredundant) modulation effects. The labels indicate the relative direction of each modulation effect; i.e., the difference in self-inhibition (VH/RH = virtual hand/real hand task; C/IC = congruent/incongruent visuo-proprioceptive mapping; Lo/Hi = low/high visibility). Note that a reduced self-inhibition implies disinhibition and increased sensitivity to inputs; i.e., an increase in gain (e.g., VH < RH indicates a stronger disinhibition during the VH than during the RH task). Most importantly, the optimized model was characterized by selective disinhibition of the V1 or S2 by the attentional set adopted in the VH task or RH task, respectively.

The aim of our DCM analysis was to identify changes in effective connectivity related to top-down modulations of post-synaptic gain by attention, motivated by a predictive coding account of brain function (Friston and Kiebel 2009). Along this account, within the cortical hierarchy, descending predictions about neural dynamics are compared against ascending (sensory) inputs and the resulting prediction error is then used to update the beliefs generating the respective predictions. At higher levels, these predictions will be multimodal—for example, about visual and proprioceptive inputs about one’s hand position—and therefore subject to (Bayesian belief) updating by prediction errors from several sensory modalities. Crucially, the relative influence of prediction error signals on belief updating can be balanced by relatively attenuating or enhancing their precision (i.e., inverse variance): a more precise prediction error signal will have a stronger impact on higher levels. Biologically, this is likely implemented by modulating the post-synaptic gain of superficial pyramidal cells encoding prediction errors—based on predictions from hierarchically higher brain areas (Bastos et al. 2012; Auksztulewicz and Friston 2015). Importantly, in predictive coding formulations, such top-down gain control corresponds to the process of attention (Feldman and Friston 2010). Indeed, several previous DCM studies using electromagnetic responses have demonstrated that attentional effects specifically target inhibitory self-connections of superficial pyramidal cell populations (Brown and Friston 2013; Auksztulewicz and Friston 2015; Adams et al. 2016; Auksztulewicz et al. 2017). In other words, these connections can be interpreted as controlling a region’s (cell population’s) gain—its input-output balance or sensitivity to inputs—as a function of attention. We expected exactly this sort of effect to underlie the BOLD signal changes observed in our experiment: a decreased self-inhibition (increased gain) in the cortical areas processing the currently attended modality and potentially an increased self-inhibition in areas processing the unattended modality.

For each participant, we specified a fully connected model (i.e., bidirectional connections between all brain regions) with driving sensory inputs entering the network at the respective “earliest” region of visual and proprioceptive information processing; i.e., the V1 and the S2 (Fig. 3). To test for attentional effects, we allowed for all possible modulations of all regional self-connections by the main effects and the significant interactions (attentional set × visuo-proprioceptive congruence and attentional set × visual salience) of our factorial design. In bilinear DCM for fMRI (Friston et al. 2003), only one cell population is modeled per region, therefore gain modulation is modeled by changes in a given region’s intrinsic (recurrent or self) connection. The full model was successfully inverted for each participant, explaining 52% of the variance on average (range: 30–64%). Moreover, it clearly outperformed an analogous model allowing for modulations of between-region connections (Supplementary Fig. 14), which further supported our hypothesis that our experimental effects would specifically target self-connections.

We used an empirical Bayesian group inversion scheme (Friston et al. 2016) to invert and compare these models, thus minimizing the effect of participant-specific uncertainty. We then used Bayesian model reduction (BMR, Friston and Penny 2011) to identify the optimal model (taking into account model complexity and accuracy) given the observed fMRI time series. BMR rests upon the inversion of a single “full” model and the subsequent evaluation of “reduced” models in terms of their model evidence. The reduced models are distinguished by the presence or absence of parameters; for example, by switching certain coupling modulations “on” or “off” (via shrinkage priors). One can therefore compare an arbitrarily large number of reduced models—for example, using a step-wise automatic search—against the full model without needing to re-fit each single model. This scheme identifies those effects that contributed to model evidence and prunes away redundant effects that did not. In other words, any retained parameter (i.e., that differs significantly from its prior) implies that a model with this modulation outperformed a model without it—and thus provides evidence for a specific modulation effect (e.g., by an experimental factor) on that connection. Here, we used BMR on a full model allowing for all self-connections to be modulated by our experimental factors to identify which of them were targeted by attentional processes. It is worth noting that only a subset of parameters were retained by the BMR, meaning that the optimized model was neither the most parsimonious nor the most complex model. Finally, to account for uncertainty about posterior parameters across a large number of similar models, we applied Bayesian model averaging (Penny et al. 2010) over the selected models within Occam’s window; i.e., weighting the parameters of each model according to the model’s log evidence.

## Results

### Behavioral results

Participants reported that the VH task was easier to perform under high visibility, whereas the RH task was easier to perform under low visibility (Wilcoxon’s signed-rank test, *Z* = 47, *P* < 0.05, see Supplementary Fig. 1). Movement amplitudes (Supplementary Table 1) did not differ between attentional sets or visibility levels (ANOVA, *F*s < 1, *p*s > 0.3), but participants made somewhat larger movements during visuo-proprioceptive incongruence (*F* = 6.75, *P* < 0.05). Participants performed better in matching the VH movements to the dot’s phase in the VH task than in the RH task, but this difference did not reach statistical significance (Supplementary Table 2). A behavioral control experiment in an independent sample (*N* = 16) confirmed that the VH and RH tasks were perceived as equally difficult (but both significantly more difficult under visuo-proprioceptive incongruence), and that participants allocated their focus of attention to the VH or RH, as instructed, during congruent and incongruent movement periods alike (Supplementary Fig. 2). In sum, these data suggest that (as expected, H1a) our task manipulated the participants’ attentional set. The eye tracking data confirmed that participants maintained central fixation in all conditions (Supplementary Fig. 4).

### Brain Activation Changes—SPM Results

The main aim of our SPM analysis was to identify brain regions that would show activation changes related to the factor attentional set (H1a). Indeed, contrasting the VH > RH task revealed a significant effect in the bilateral SPL (L. Brodmann area (BA) 5 L/R. BA 7A; *P* < 0.05, corrected for multiple comparisons, Fig. 2*A*). At more liberal (uncorrected) statistical thresholds, further notable task effects were found in the left V5 and V1 (see Supplementary Figs 7 and 8). The obtained activations in the contralateral SPL and V5 replicated key findings from our recent fMRI study using a similar virtual hand target tracking task (Fig. 11; cf. Limanowski et al. 2017). Furthermore, as expected, the converse contrast RH > VH task revealed a significant effect in the left S2 (area OP1; *P* < 0.05, corrected, Fig. 2*A*). Periods of incongruent > congruent visuo-proprioceptive hand movement were associated with significantly increased responses in bilateral posterior temporal regions and in the left S2 (null conjunction contrast, *P* < 0.05, corrected, Fig. 2*B*; the reverse contrast revealed significant activations in the right putamen). VS (high > low visibility) was reflected by significantly increased responses in the bilateral occipital lobe, centered on the left V1 (spanning to extrastriate cortices) and in the hippocampus; the reverse contrast showed significant effects in the cuneus and temporo-parietal regions (Fig. 2*C*). Furthermore, the left SPL showed a significant interaction effect between attentional set and visuo-proprioceptive congruence: a relatively larger response increase during periods of visuo-proprioceptive incongruence in the VH > RH task (Supplementary Fig. 9). The left S2 showed a significant interaction effect between attentional set and visual salience: a relatively larger activation difference between low > high visibility conditions during the RH > VH task set. See Table 1 and Supplementary Figures 6–9 for details. The interaction between visuo-proprioceptive congruence and visual salience, as well as the three-way interaction yielded no significant effects (see Supplementary Fig. 10 for uncorrected effects). In sum, the main effects and interactions engaged brain regions that have been established as part of the functional anatomy of sensorimotor integration in this sort of paradigm, in line with our hypotheses (H1b and H1c) based upon set dependent precision weighting and attentional gain.

**Table 1:** Significant (*P* < 0.05, FWE corrected for multiple comparisons) activations of all reported contrasts

Anatomical location	Voxels		MNI (*x*, *y*, *z*)		Peak *T*	Peak *p*_FWE_
Attentional set VH > RH						
L. SPL (BA 5L)	24	−32	−44	56	6.14	0.006
R. SPL (BA 7M)	18	22	−60	54	5.77	0.018
R. SPL (IPS)	1	26	−50	48	5.44	0.046
Attentional set RH > VH						
L. S2 (OP1)	6	−56	−28	14	4.65	0.012^a^
Visual salience Hi > Lo						
L./R. V1, V3, V4, and V5	5335	−12	−96	0	24.63	<0.001
L. Hippocampus	30	24	−30	−6	7.25	<0.001
R. Hippocampus	12	−20	−32	−2	6.47	0.002
Visual salience Lo > Hi						
L. Cuneus (V3d)	193	2	−84	24	7.38	<0.001
L. Inferior temporal gyrus	14	−62	−58	−6	6.26	0.004
R. Inferior parietal lobe/angular gyrus	14	44	−74	36	5.81	0.016
L. Inferior parietal lobe/angular gyrus	47	−44	−78	34	5.76	0.018
Visuo-proprioceptive congruence IC > C (null conjunction across VH Hi, VH Lo, RH Hi, and RH Lo)						
L. Superior temporal gyrus and S2 (OP1)	97	−48	−36	14	7.39	<0.001
R. Superior temporal gyrus/sulcus	123	60	−30	18	6.51	0.002
R. Superior temporal gyrus/sulcus	31	−62	−40	8	6.13	0.007
R. Middle temporal gyrus	3	46	−48	6	5.81	0.018
L. Postcentral gyrus/rolandic operculum (S2)	4	−64	−4	12	5.60	0.033
R. Middle temporal gyrus	2	44	−66	4	5.56	0.037
L. S2 (OP4)	1	−62	−12	14	5.51	0.043
L. Middle temporal gyrus	1	−58	−34	6	5.46	0.049
Visuo-proprioceptive congruence C > IC (null conjunction across VH Hi, VH Lo, RH Hi, and RH Lo)						
R. Putamen	2	24	4	6	5.97	0.011
Interaction attentional set × visuo-proprioceptive congruence (VH IC–VH C) > (RH IC–RH C)						
L. SPL (BA 5L)	2	−32	−44	58	2.71	0.047^b^
Interaction attentional set × visual salience (VH Hi–VH Lo) > (RH Hi–RH Lo)						
L. S2 (OP1)	6	−56	−26	14	3.72	0.001^b^
L. Area TE_3_	1	−64	−12	6	5.42	0.048
Interaction attentional set × visual salience (RH Hi–RH Lo) > (VH Hi–VH Lo)						
R. Parahippocampal gyrus	1	24	−22	−26	5.60	0.029

^**a**^FWE correction within an anatomical mask comprising the left somatosensory cortices (areas BA 1, 2, 3b, and OP 1–4).

^**b**^FWE correction within areas showing a significant main effect of attentional set.

### Changes in Neuronal Gain—DCM Results

Next, we asked whether the activation differences identified by our SPM analysis could be explained in terms of gain modulations of neuronal populations in the respective brain regions using DCM. This analysis was motivated by our hypothesis (H1a) that participants would adopt a specific attentional set to comply with the task instructions, which would manifest itself as changes in regional gain or self-inhibition—as modeled with DCM (H2). We defined a fully connected model comprising the left V1, V5, S2, and SPL, with sensory inputs driving the respective lowest visual (V1) and proprioceptive (S2) regions. We allowed for modulations of all intrinsic regional self-connections (which encode self-inhibition and thus model post-synaptic gain) by all experimental main effects and significant interactions. We then used BMR to determine which of the model’s parameters were necessary to explain the observed BOLD signal time series—in other words, we determined which regions (if any) would show gain modulations of the sort associated with attention related to our experimental factors.

The results of this analysis (Fig. 3, cf. Supplementary Fig. 15) revealed evidence for several modulations of regional self-inhibition at specific levels of the network: Most importantly, attentional set was mediated by diametrically differential gain modulation of the V1 and the S2; the V1 was disinhibited under the VH task, while the S2 was disinhibited under the RH task. This means that the effects of the adopted attentional set could be recovered in terms of selective gain modulations in the respective sensory areas processing the task-relevant modality. Furthermore, visuo-proprioceptive incongruence was associated with a relative disinhibition of the S2. visual salience disinhibited the V1, while increasing the inhibition of the V5 and the SPL. There was further evidence for a modulation of the S2 by the interaction between attentional set and visuo-proprioceptive congruence; the S2 was relatively more strongly disinhibited by visuo-proprioceptive incongruence during the VH task than during the RH task. No other interaction effects were retained by the BMR, meaning that they did not contribute to model evidence. This is unsurprising because neuronal interactions can usually be well captured by modulations by the experimental main effects (Auksztulewicz and Friston 2015).

## Discussion

We used a virtual reality environment to investigate whether endogenous attention (instructed via task-relevance) can change the weighting of visual versus proprioceptive hand movement cues during action. Our main finding was that the attentional set adopted by our participants (confirming H1a) was reflected by diametrical activity changes in visual, proprioceptive, and multisensory brain regions contralateral to the moving hand (confirming H1b), which could be modeled as resulting from top-down gain modulations of sensory processing (confirming H2).

Our SPM analysis showed that the SPL generally increased its activity during the VH task (similar but weaker effects were found in the V1 and V5), whereas the S2 increased its activity during the RH task. These results suggest that the left SPL was accumulating task-relevant visual hand movement information into a multisensory body representation for action control; its additional activity modulation by visuo-proprioceptive incongruence supports previous suggestions that the SPL resolves intersensory conflicts to maintain a single multisensory body representation (Wolpert et al. 1998; Graziano et al. 2000; Grefkes et al. 2004; Ogawa et al. 2006; Limanowski and Blankenburg 2015a, 2016, 2017). Conversely, we propose that the increased S2 responses during the RH task reflected proprioceptive attention (i.e., attention to the real, unseen hand), which fits well with previous studies implicating the S2 in “motor” attention (Rushworth et al. 2001; cf. Rowe et al. 2002) or somatosensory awareness (Romo and Salinas 2001; Auksztulewicz et al. 2012; Limanowski et al. 2019). Moreover, we found that visuo-proprioceptive incongruence was associated with relatively increased activity of bilateral posterior superior and middle temporal regions. These findings speak to previous proposals that a domain general function of these areas is to detect mismatches between executed and observed actions (Leube et al. 2003; Farrer et al. 2008; Limanowski, Sarasso et al. 2018; van Kemenade et al. 2019) and a correspondingly general attenuation (or “cancellation”, cf. Ghahramani et al. 1997; Straube et al. 2017) of sensory reafference.

The DCM analysis showed that V1 gain was increased under the VH task, while S2 gain was increased under the RH task. These results suggest that a task-induced attentional focus on the visual or proprioceptive modality increased the gain of neuronal populations in sensory areas processing the respective attended modality relative to those processing the irrelevant, “ignored” modality. We propose that the increased V1 gain allowed visual information to dominate over proprioception, resulting in its stronger integration into the body representation for action. We speculate that these integration processes took place in the SPL. This interpretation speaks to the reported benefits of the temporary suspension or attenuation of somatosensory information processing when adapting to novel visuo-motor mappings (Taub and Goldberg 1974; Ingram et al. 2000; Balslev et al. 2004; Bernier et al. 2009). Conversely, the increased S2 gain was likely a manifestation of proprioceptive attention, which also explains the related activation increases during the RH > VH task observed in the SPM analysis.

Furthermore, visuo-proprioceptive incongruence was associated with increased activity in the S2—which was reflected in terms of a relative increase in regional gain in the DCM analysis. Given that both tasks were perceived as more difficult under visuo-proprioceptive incongruence (cf. Supplementary Fig. 8), this supports the assumption that the S2 was generally activated by motor or proprioceptive attention. Interestingly, there was some evidence for that this effect was more pronounced during the VH task (i.e., the modulation of S2 gain by the interaction between attentional set and visuo-proprioceptive congruence). This nicely reflects the fact that visuo-proprioceptive recalibration in fact also requires a considerable amount of attention to one’s movements—in the sense that these need to conform to the novel visuo-motor mapping.

Finally, we found increased V1 responses and gain during high visibility, which supports previous findings reporting salience or contrast gain effects in V1 (Gardner et al. 2005; Brown and Friston 2012). Conversely, V5 and SPL gain increased during low visibility. This may suggest a more or less “automatic” increase of attention to visual motion (Büchel and Friston 1997) in order to compensate for the loss of visual precision—this phenomenon has also been seen in the DCM of slow visual pursuit of a sinusoidal target (Adams et al. 2015).

Our results advance previous work by showing that during multisensory integration for action, the weights assigned to each sensory modality are not only determined by sensory precision (e.g., Ernst and Banks 2002; Beauchamp et al. 2010) but can also be mediated by endogenous attention. This finding can potentially help to understand a key mechanism of self-other distinction in action execution versus observation: within the predictive coding framework, the relative balance between visual and proprioceptive prediction errors determines inference about whether “I am moving” or whether “I am observing an action” (^50,51^; Friston 2012; cf. Brass et al. 2001). Moreover, the fact that people can deliberately influence how visual information is integrated into their body representation may help understand and develop immersive experiences such as virtual reality applications involving avatars or artificial limbs (cf. Carmena et al. 2003; Metzinger 2007; Press 2011).

We interpret the observed modulations of regional self-inhibition within predictive coding theories of brain function, where these modulations imply top-down gain control or attention; i.e., a mechanism by which the influence of ascending prediction error signals can be selectively attenuated or enhanced through modulations of post-synaptic gain of neuronal populations based on predictions of precision from hierarchically higher brain areas (Feldman and Friston 2010; Auksztulewicz and Friston 2015; cf. Thiele and Bellgrove 2018). In this light, our results beg the question: which brain areas are the source of these attentional modulations? Notably, we found (uncorrected) activity modulations by attentional set—and/or its interaction with visuo-proprioceptive congruence—in several areas that have been specifically linked to top-down attentional control (Supplementary Figs 7 and 10): the SPL (Hopfinger et al. 2000; Blankenburg et al. 2010), the premotor cortex (Ruff et al. 2006), the dorsolateral prefrontal cortex (Fink et al. 1999; Rowe et al. 2002; Gazzaley and Nobre 2012), the thalamus (Saalmann and Kastner 2011), and the cerebellum (Allen et al. 1997; cf. Straube et al. 2017; van Kemenade et al. 2019). All of these are plausible candidates for brain areas issuing the corresponding predictions of precision. However, bilinear DCM for fMRI does not model the sources of top-down modulations. In our future work, we will try to answer this question using magnetoencephalography together with more detailed neurobiological models for DCM. These models will allow tests for the specific assumptions of predictive coding, by identifying the specific neuronal population targeted by attentional mechanisms. This can be established by using Bayesian model comparison to identify activity-dependent changes in “forward” and “backward” connectivity, or by including non-linear modulations of one neuronal population’s gain by another. Other important questions for future work include potential grasping phase-dependent neuronal responses in visual versus somatosensory areas during visuo-proprioceptive incongruence, or how one could more effectively introduce visual and proprioceptive noise (introduced e.g., via tendon vibration, Jaeger et al. 1979) in similar paradigms.

One potential limitation of our study is that visuo-motor recalibration (i.e., adjusting one’s movements to keep the VH phase-matched with the dot) was not significantly better in the VH than in the RH task; although showing a tendency in the expected direction. The high task difficulty (as reported by most of our participants) might explain the relatively small differences compared to previous work using easier tasks (such as tracking a moving visual target with a mouse or joystick cursor, e.g., Ogawa et al. 2006). One reason for the non-significant difference was that participants partly exhibited visuo-motor adaptation in the RH task, too (cf. Supplementary Table 2). These results could partly be explained by an “automatic imitation” of observed movements (cf. Kilner et al. 2003, 2007). We will pursue this idea in our future work. Nevertheless, in sum our behavioral and questionnaire results suggest that participants tried to comply with the instructions and that this sufficed to engage the attentional processes of interest.

To conclude, our results suggest that, during action, attention can modulate visuo-proprioceptive gain to adjust the relative influence of each modality on inferences about body state in multisensory brain regions. This attentional selection can augment or attenuate the integration of visual movement cues into the own body representation, depending on the current behavioral context.

## Funding

The European Union’s Horizon 2020 Research and Innovation Programme under the Marie Skłodowska-Curie (grant agreement 749988 to J.L.); Wellcome Trust Principal Research Fellowship (Ref: 088130/Z/09/Z to K.F.).

## Author Contributions

J.L. and K.F. designed study; J.L. acquired and analyzed data; J.L. wrote manuscript; K.F. commented on the manuscript.

## Notes

We would like to thank Felix Blankenburg for providing the data glove, and Peter Zeidman for helpful comments on the DCM analysis. *Conflict of Interest*: The authors declare no conflict of interest.

## Supplementary Material

LimanowskiFriston_SupplementaryMaterial_bhz192

Limanowskietal_VideoExample_bhz192
