# Supplementary material for: Attentional Modulation of Vision Versus Proprioception During Action
Source: Cereb Cortex. 2019 Oct 16;30(3):1637–48. doi: 10.1093/cercor/bhz192 (PMC7132949; doi:10.1093/cercor/bhz192)
Supplement: LimanowskiFriston_SupplementaryMaterial_bhz192 [file limanowskifriston_supplementarymaterial_bhz192.pdf]

Supplementary material

Attentional modulation of vision vs proprioception during action, J Limanowski and K Friston

Figure S1. Participants' mean post-scanning ratings of task difficulty depending on the visibility of the virtual hand (with associated standard errors of the mean). As expected, participants found the virtual hand easier under high visibility of the virtual hand, and the real hand task easier under low visibility (Wilcoxon's signed-rank test, $Z = 47$, $p < 0.05$). VH/RH = virtual hand/real hand task.

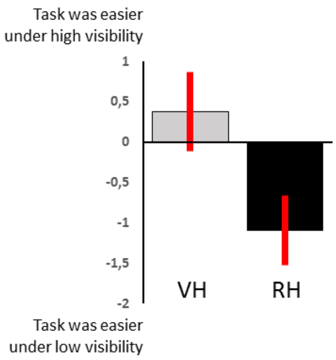

Table S1. Mean movement amplitudes (with associated standard deviations) for each cell in the factorial design. Movement amplitudes did not differ between attentional sets or visibility levels (ANOVA, *F*s < 1, *p*s > 0.3), but participants made somewhat larger movements during visuo-proprioceptive incongruence ($F = 6.75$, $p < 0.05$); none of the interaction effects was significant (all *F*s < 1.7, *p*s > 0.2). VH/RH = virtual hand/real hand task; C/IC = congruent/incongruent visuo-proprioceptive movement; Lo/Hi = low/high visibility.

	**VH Hi**	**VH Lo**	**RH Hi**	**RH Lo**
**C**	0.77 *(0.09)*	0.79 *(0.07)*	0.80 *(0.08)*	0.81 *(0.07)*
**IC**	0.82 *(0.06)*	0.80 *(0.08)*	0.82 *(0.08)*	0.83 *(0.07)*

Table S2. Mean phase matching performance (with associated standard deviations) during visuo-proprioceptive incongruence. The values indicate the relative difference between the phase of the pulsating fixation dot and the phase of the *virtual* hand's movements. Under the virtual hand task instruction, participants were more accurate at matching the virtual hand to the dot's phase, but this difference was not statistically significant (non-parametric Friedman's test, $\chi^2 = 2.02$, $p = 0.57$). VH/RH = virtual hand/real hand task; Lo/Hi = low/high visibility.

**VH Hi**	**VH Lo**	**RH Hi**	**RH Lo**
134.9 *(17.2)*	141.2 *(17.0)*	140.6 *(27.7)*	141.7 *(24.6)*

Figure S2. We conducted a behavioral control experiment on a separate sample of 16 participants (9 female, mean age = 26.6 years, range = 21-31 years) that had not participated in the fMRI experiment, using a virtually identical task as used in the fMRI experiment – for clearer perceptual categorization, we did not manipulate visual salience and we presented congruent and incongruent conditions separately. I.e., the four conditions 'virtual hand task under congruence' (VHc), 'virtual hand task under incongruence' (VHic), 'real hand task under congruence' (RHc), and 'real hand task under incongruence' (RHic) were presented 3 times per run each in randomized order; the delay was set to 500 ms. Participants completed a brief training, and two 8-minute runs of this task, after signing informed consent.

After the experiment, participants were asked to indicate – for each condition separately, on a 7-point visual analog scale – their answers to the following two questions: "How difficult did you find the task to perform in the following conditions?" (Scale from "very easy" to "very difficult") and "On which hand did you focus your attention while performing the task?" (Scale from "I focused on my real hand" to "I focused on the virtual hand").

For the ratings of the first question, a nonparametric Friedman's test (due to non-normal distribution of the data) revealed a significant difference between conditions (χ^2 = 38.72, p = 0.00000002), with incongruent conditions being rated more difficult than congruent conditions. Post-hoc comparisons using Wilcoxon's signed rank test confirmed that VHic > VHc (p = 0.00006), RHic > RHc (p = 0.00006), but no difference between VHic vs RHic (p = 0.25) or VHc vs RHc (p = 0.25). These results suggest that, in line with our assumptions, the virtual hand and the real hand task were overall perceived as equally difficult, and that in both cases the added incongruence increased task difficulty. Tentatively, this means that our task was not substantially biased towards either modality.

For the ratings of the second question, a nonparametric Friedman's test (due to non-normal distribution of the data) revealed a significant difference between conditions (χ^2 = 31.73, p = 0.0000006), i.e., participants focused more strongly on the virtual hand during the virtual hand task, and more strongly on the real hand during the real hand task. Post-hoc comparisons using Wilcoxon's signed rank test confirmed that this was the case for the congruent and incongruent virtual hand tasks (VHc > RHc, p = 0.0002; VHic > RHic, p = 0.0002), but there was no difference between VHic vs VHc (p = 0.84) or RHic vs RHc (p = 0.96). These results show that participants focused their attention on the instructed target modality irrespective of whether the current movement block was congruent or incongruent. This supports our assumption that participants would adopt a specific attentional set throughout each movement block – depending on the instructed target modality.

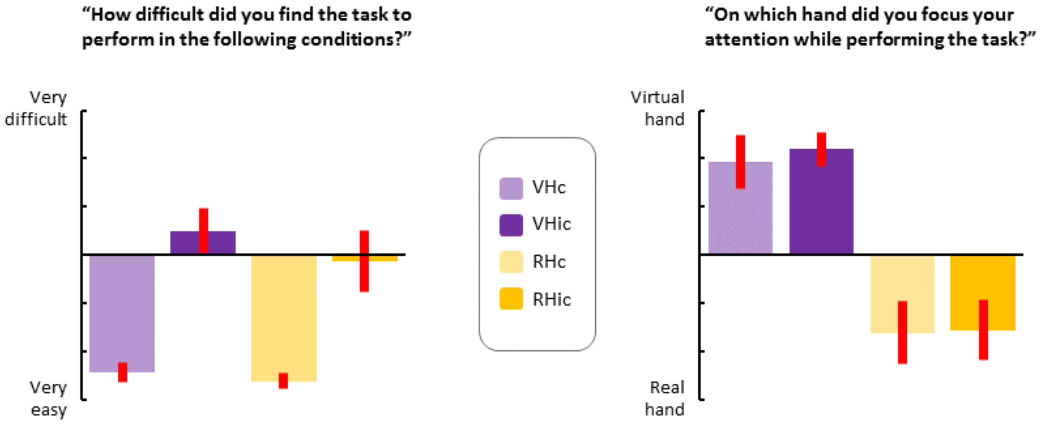

Figure S3. Averaged normalized hand movement trajectories of our participants (dotted black lines) in each experimental condition, relative to the oscillatory phase of the fixation dot (thick grey line). The plots show that participants were able to pace their grasping movements according to the fixation dot's frequency, within reasonable limits.

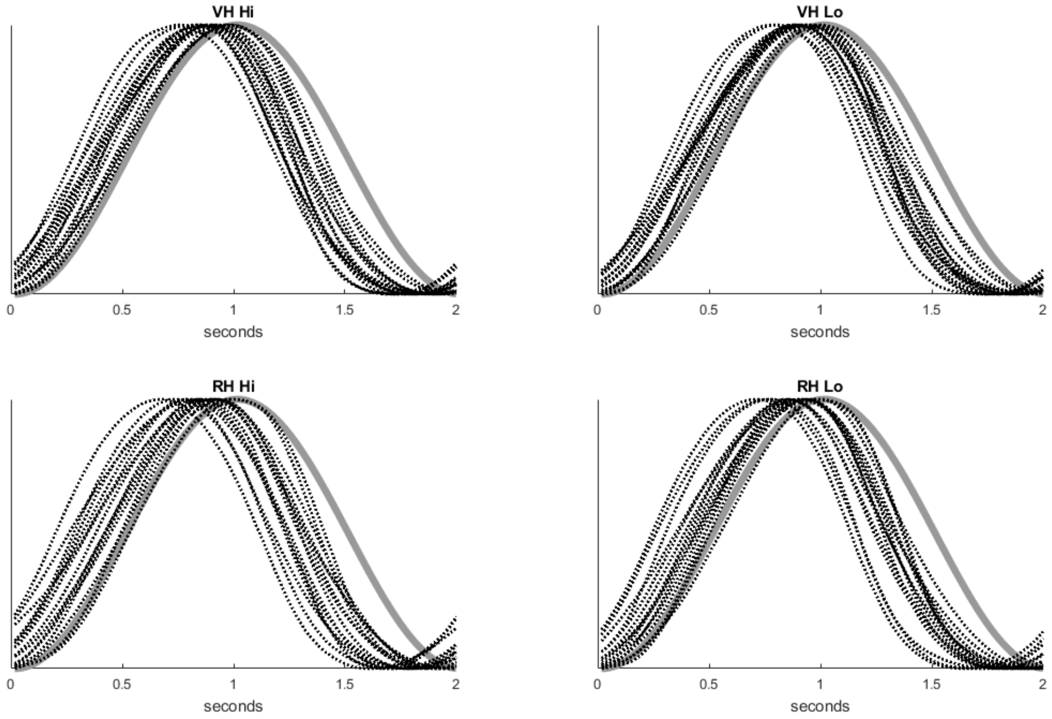

Figure S4. We monitored the participants' eye position in real time to verify that they maintained central fixation. Here, as a proof of concept, we plot the recorded eye traces (in pixels; relative to baseline fixation, marked by a red cross at [0,0]) across all runs of 12 participants (data from 4 participants had to be excluded due to unsuccessful eye tracking in the scanner) for each cell of the factorial design, after removal of blinks and periods during which pupil position was lost by the eye tracker.

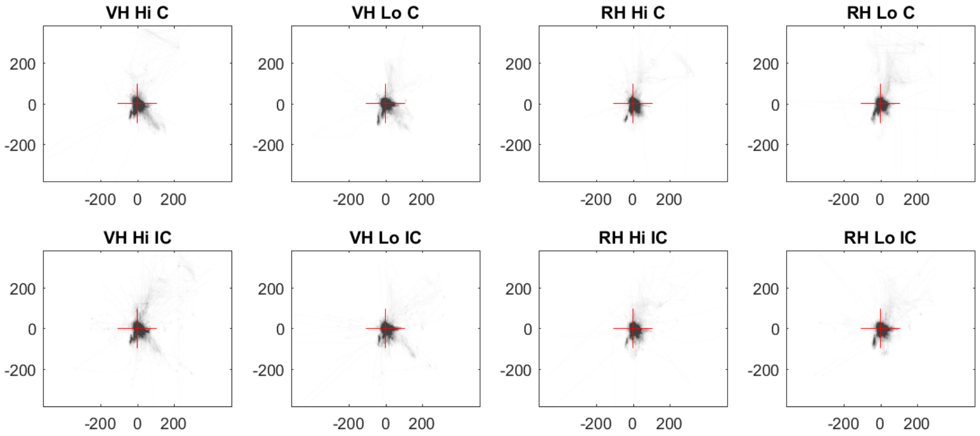

Figure S5. Example of a design matrix used in our study. Participants moved in blocks of 32 s duration under two different instructions (matching the virtual hand, VH, or the real hand, RH, to the pulsation phase of the target; i.e., factor 'attentional set') and two different contrast levels (high, Hi, or low, Lo; i.e., factor 'visual salience'). These movement blocks were modelled as 32 s long block regressors for each condition (VH_Hi, VH_Lo, RH_Hi, RH_Lo), here labelled in green. The third factor of our design 'visuo-proprioceptive congruence' was a nested factor, i.e., the virtual hand movements were congruent during the first half of each movement block and incongruent during the second half. This was modelled as a parametric modulation of each block regressor, i.e., a regressor encoding the first half of each block with -1 and the second half with +1. The block and parametric modulation regressors were entered into two separate, equivalent group-level designs with the factors attentional set and visual salience, as schematically shown below.

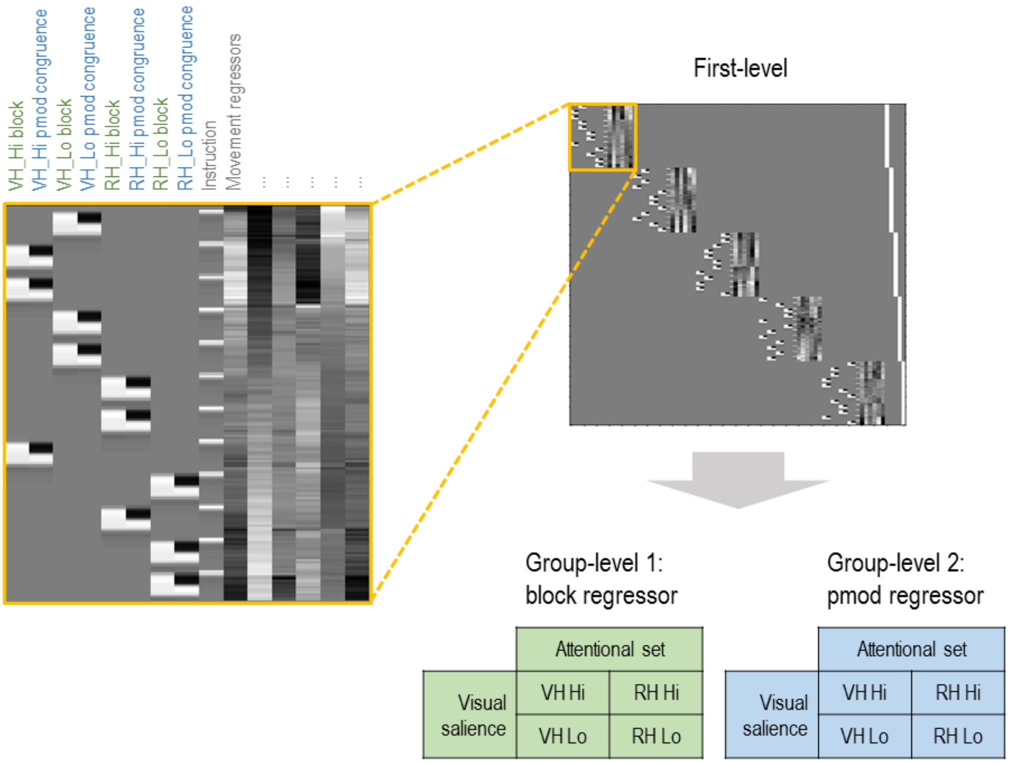

Table S3. We calculated the correlation coefficients (for each run and participant) between each of the experimental conditions and the six head movement regressors as included in the first-level GLMs. On average, none of the conditions was systematically correlated with any head movement regressor (mean r = -0.003, SD = 0.21). The average correlation coefficients (with associated standard deviations) for each condition and the associated six head movement parameters are listed below. Importantly, differences in these correlations between conditions were not significant (all *p*s > 0.11). This suggests that potentially task-related head movements were not systematically different between conditions.

	x	y	z	pitch	roll	yaw
VH Hi	0.03 (0.23)	0.02 (0.21)	-0.02 (0.22)	0.01 (0.23)	0.02 (0.23)	0.01 (0.23)
VH Lo	-0.01 (0.22)	0.01 (0.20)	0.01 (0.20)	-0.04 (019)	-0.03 (0.22)	-0.03 (0.23)
RH Hi	-0.06 (0.21)	0.03 (0.17)	0.02 (0.19)	-0.01 (0.20)	-0.02 (0.19)	-0.04 (0.22)
RH Lo	-0.01 (0.21)	-0.02 (0.21)	0.02 (0.19)	0.00 (0.18)	0.03 (0.23)	0.00 (0.20)

Figure S6. Render of significant ($p < 0.05$, corrected for multiple comparisons) activations obtained by contrasting all movement blocks > rest periods.

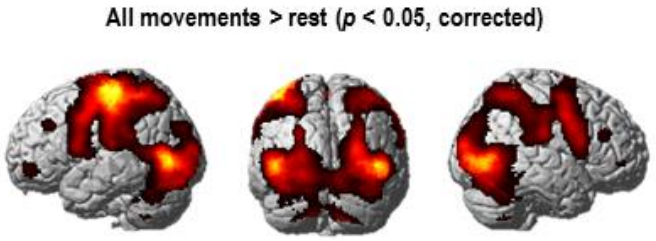

Figure S7. Activation differences between the virtual hand and real hand tasks (displayed at $p < 0.001$, uncorrected for multiple comparisons). Besides the bilateral posterior parietal cortex, the virtual hand task also engaged areas in the bilateral premotor cortex, posterior intraparietal sulcus, and V5.

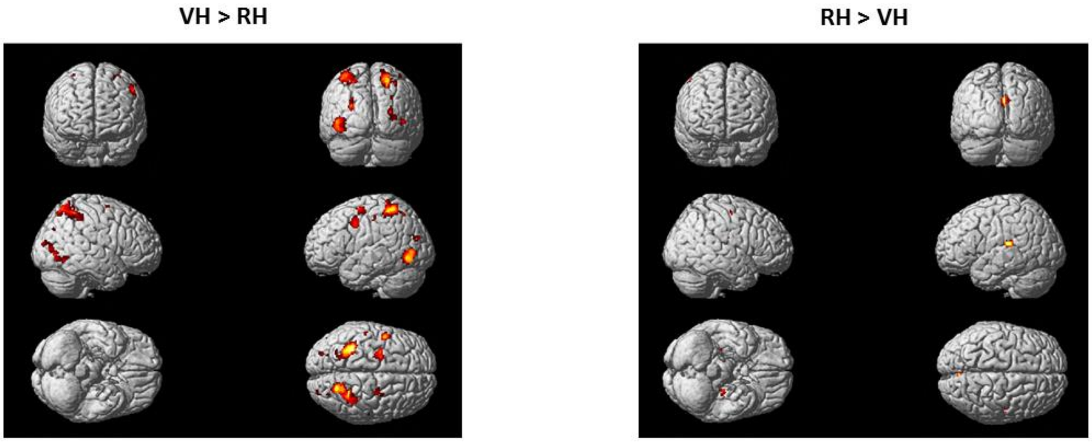

Figure S8. Stronger activation of the left V5 and V1 during the virtual hand task (VH > RH; V5 activation displayed at $p < 0.001$, uncorrected; V1 activation displayed at $p < 0.05$, uncorrected, within an anatomical mask of left and right hOc1/V1). The bar plots show the contrast estimates from the individual participants' peaks within 10 mm of the group maximum. Note that the left V1, besides showing a strong effect of visual salience (Hi > Lo) also showed generally stronger activation during the VH > RH task.

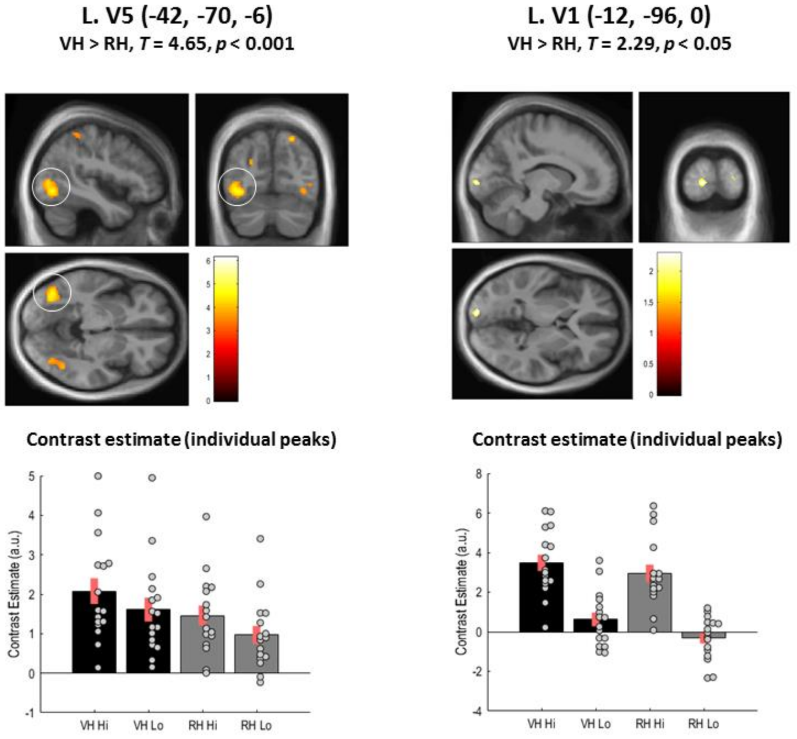

Figure S9. SPMs of significant ($p < 0.05$, corrected for multiple comparisons) interaction effects between attentional set and visuo-proprioceptive congruence in the left SPL (A) and left S2 (B). Here the contrast estimates reflect the strength of the parametric modulation by incongruent > congruent movement periods in each movement block. VH/RH = virtual hand/real hand task; C/IC = congruent/incongruent visuo-proprioceptive movement; Lo/Hi = low/high visibility.

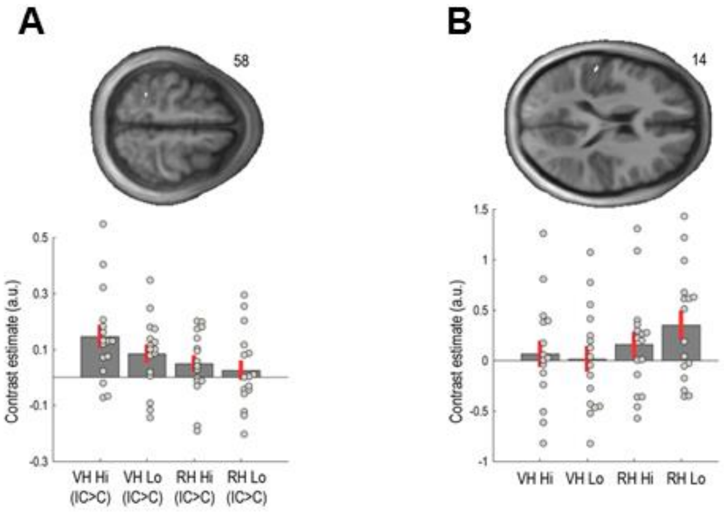

Figure S10. Activations obtained from T-contrasts testing for all interaction effects ($p < 0.001$, uncorrected for multiple comparisons).

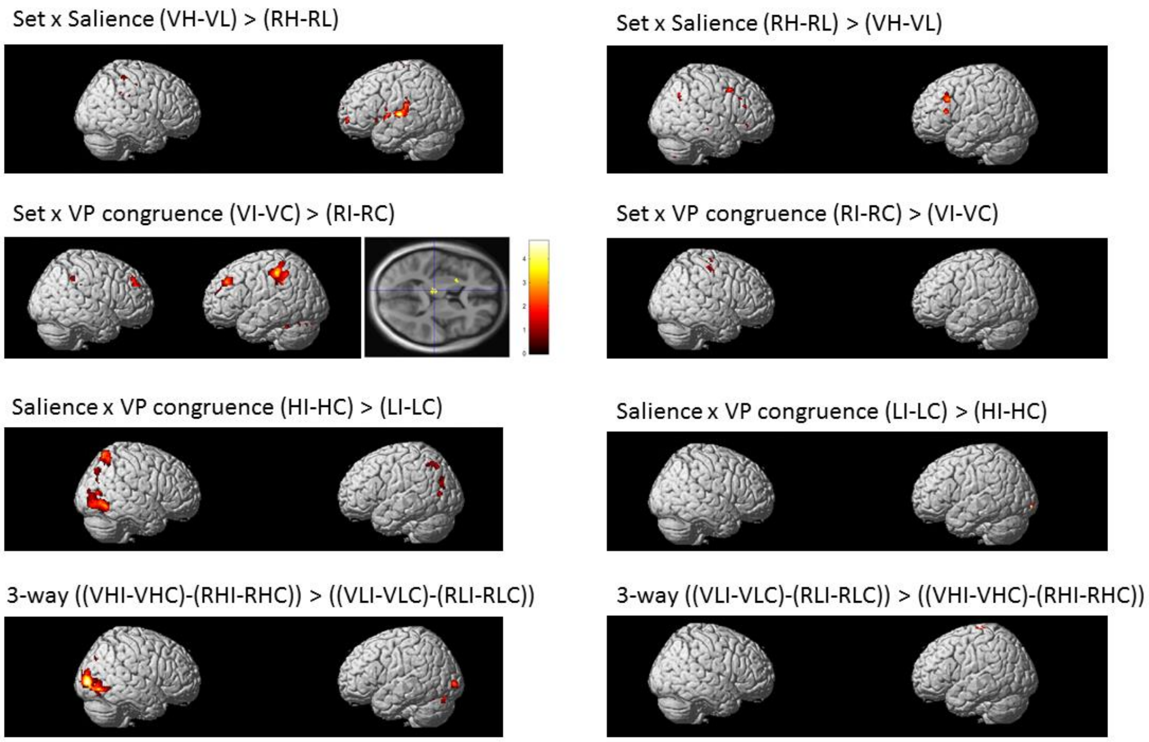

Figure S11. Replication of the results from a recent study (Limanowski et al., 2017), in which we used a similar target tracking task, but tracking a moving target and, importantly, only under the instruction to track the target with the virtual hand (i.e., similar to the 'virtual hand' task in the present study; this contrast yielded significant ($p < 0.05$, corrected for multiple comparisons) activations in the left SPL ($T = 4.85$) and the left V5 ($T = 4.83$)). On the left side of the figure, we show the SPM resulting from the VH > RH contrast in the present study (thresholded at $p < 0.001$, uncorrected), the SPM result from the corresponding contrast (target tracking) from Limanowski et al. (2017), and the overlap, i.e.: voxels obtained from the VH > RH contrast in the present study that were significant at $p < 0.05$, corrected for multiple comparisons within the areas activated by the contrast from Limanowski et al. (2017; cf. middle panel). These notably include the left SPL and V5; i.e., activation differences for the VH > RH task in these regions were significant ($p < 0.05$, corrected) when restricting the search space to regions showing significant effects in a similar task in our previous study. Analogously, on the right side of the figure, we show the results of the present study, of the corresponding contrast from Limanowski et al. (2017), as well as the ($p < 0.05$, corrected) overlap for the contrast looking for activations related to visuo-proprioceptive incongruence. These included bilateral temporoparietal regions (superior temporal gyrus and sulcus).

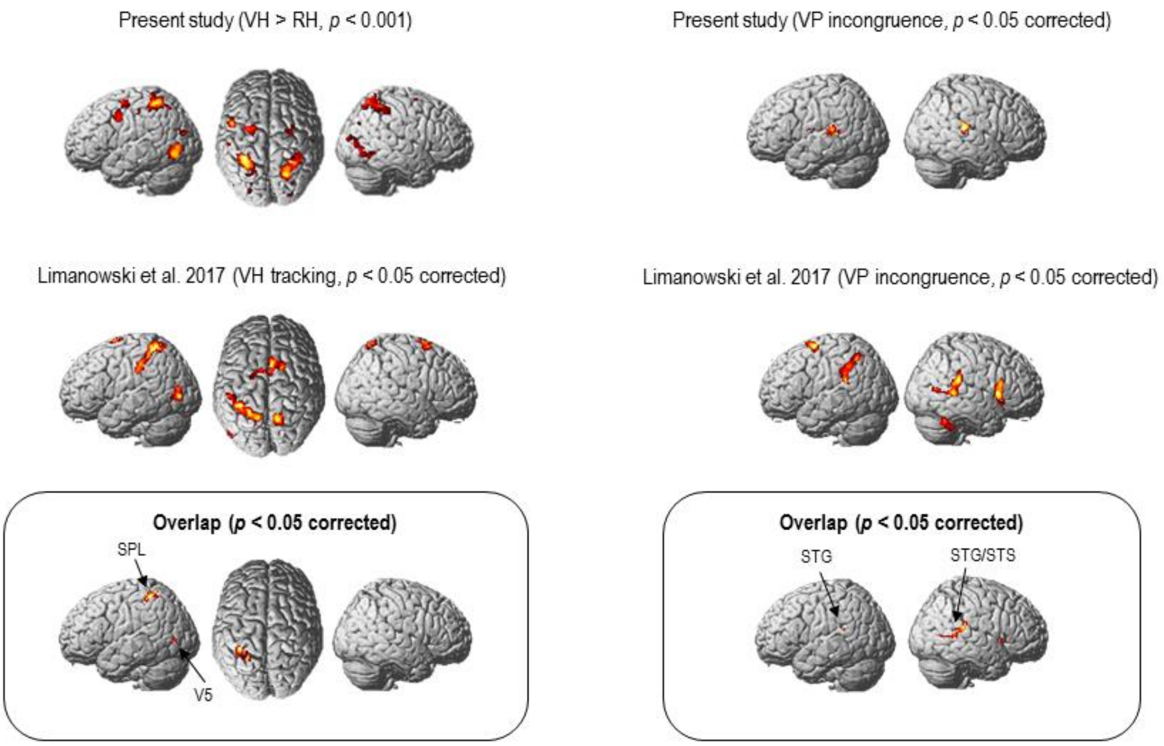

Figure S12. For time series extraction, we constructed new first-level design matrices in which the 5 runs of each participant were concatenated into one run (both design matrices are shown after estimation, i.e., the regressors have been convoluted with the hemodynamic response function). An example of an extracted time series (adjusted for regressors of no interest and session means) across all scans of one participant is shown below.

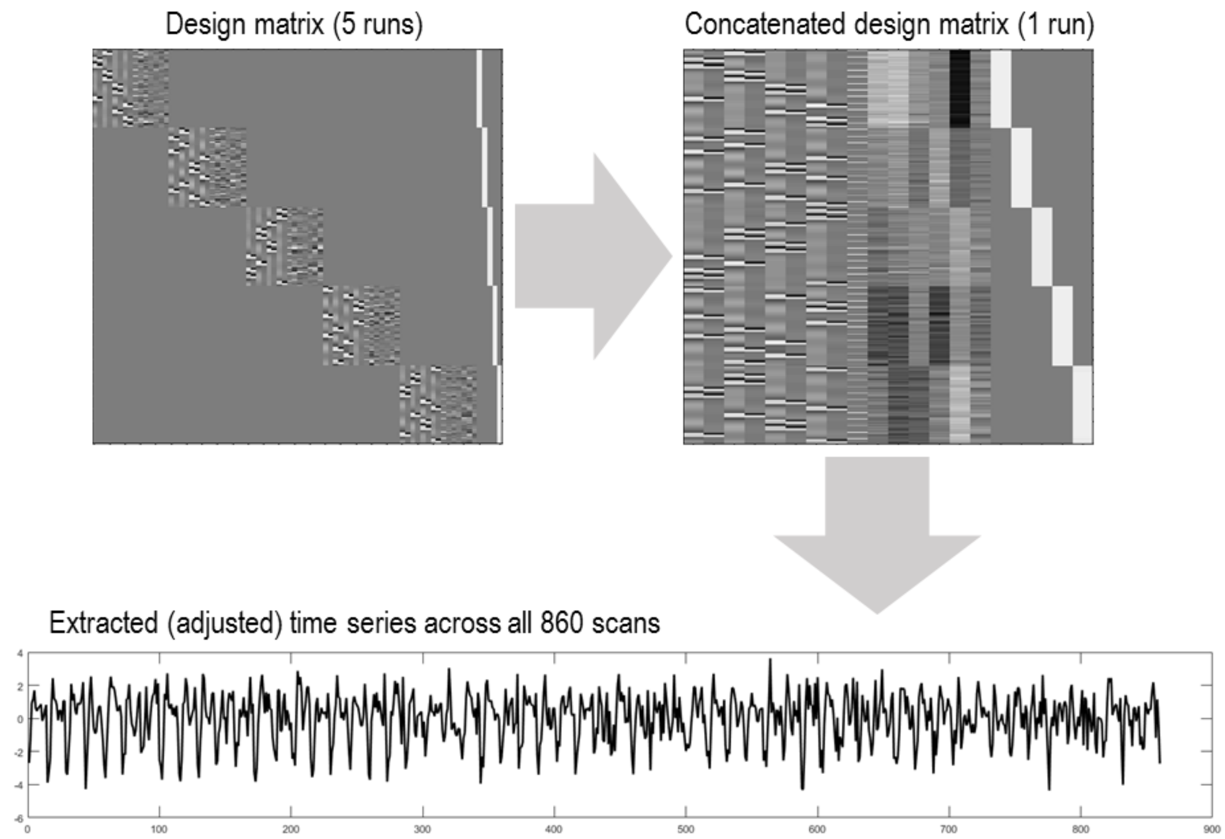

Figure S13. Example of a DCM design matrix specifying the driving and modulatory inputs to the model. Driving inputs were defined as all movement blocks (1). This regressor was modelling sensory (i.e., visual and somatosensory) inputs during the entire 32 s long movement block. Modulatory inputs were defined as main effects and interactions, i.e., encoded via 1/-1. For example, the main effect of attentional set was modelled by a regressor set to 1 for all 'virtual hand' movement blocks and -1 for all 'real hand' movement blocks. The resulting parameter estimates can therefore be interpreted as relative differences in modulation, e.g., a stronger modulation of a particular connection by the 'virtual hand' task than the 'real hand' task. Only interactions that yielded significant SPM results were included.

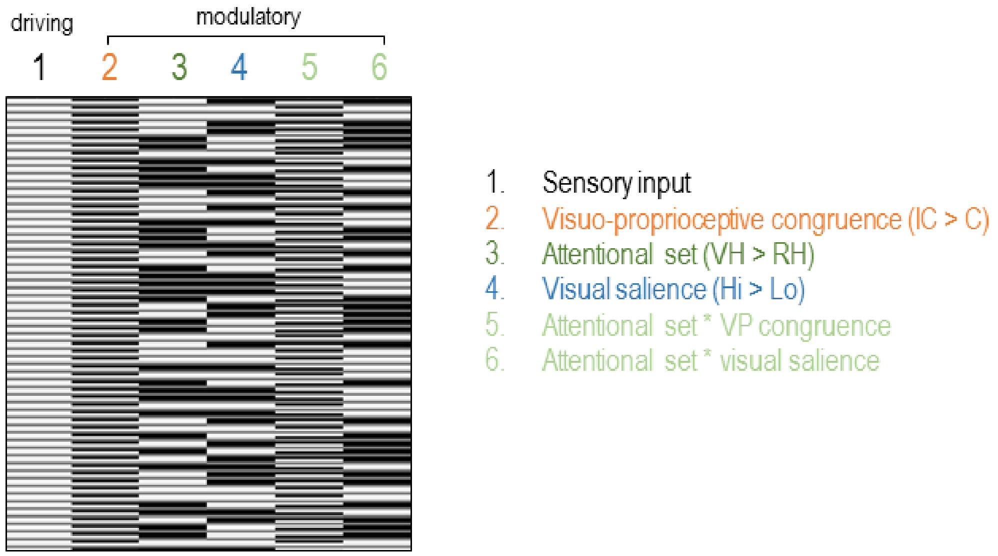

Figure S14. Our main motivation for using DCM was to identify which self-connections were the subject of attentional modulation—*that* attention operates on self- rather than between-region connections has been shown in many previous DCM studies (Brown & Friston, 2013; Auksztulewicz & Friston 2015; Adams et al., 2016; Auksztulewicz et al., 2017). As a supplementary analysis, we used Bayesian model comparison to evaluate DCMs allowing for modulations of intrinsic, i.e., self-connections ('INTR') vs extrinsic, i.e., between-region connections ('EXTR'). The INTR model had a much higher free energy than the EXTR model (the difference in free energy was 4675.9) and was clearly selected as the more likely model, with a posterior probability of 1.

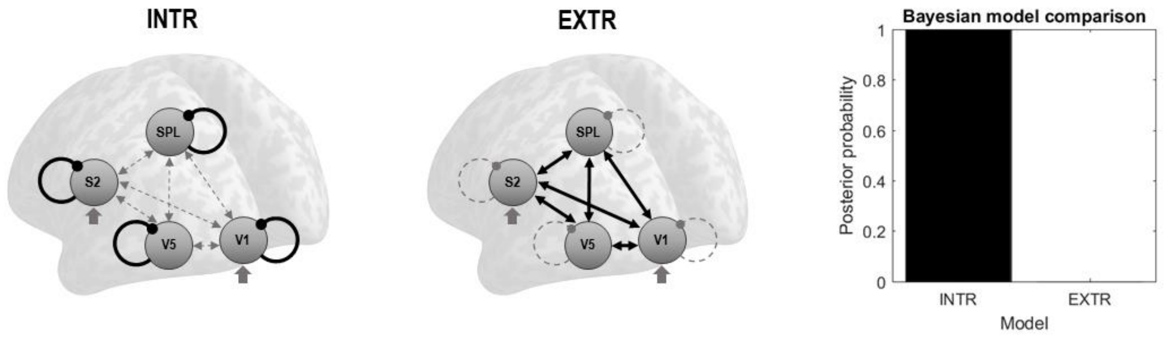

Figure S15. Plots showing the results of the final iteration of the automatic search used for BMR; i.e., the log-posterior and model posterior probabilities of the 128 most likely models; the associated model space; and the full and reduced models' parameters with associated parameter posterior probabilities.

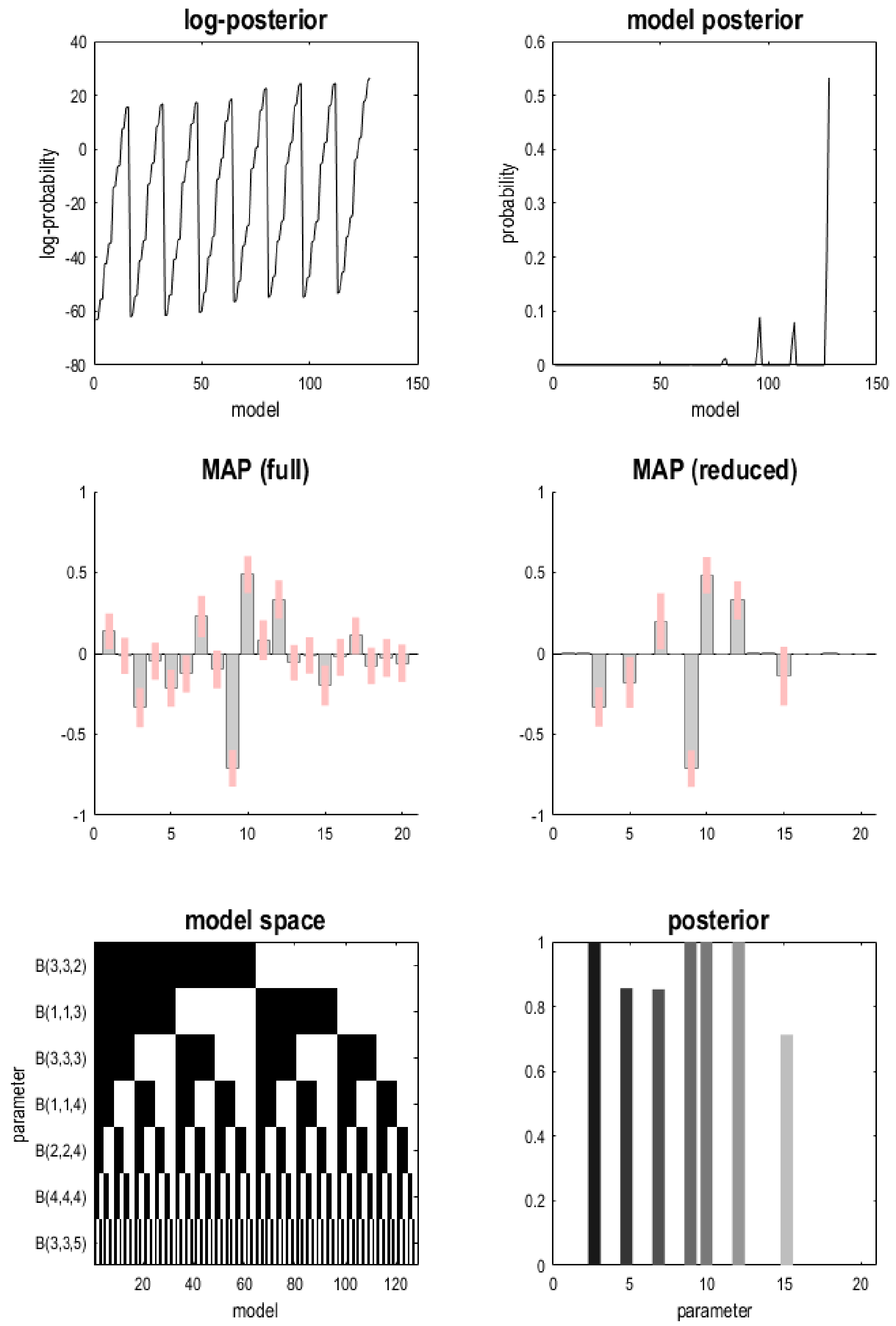

Supplementary references

Adams, R. A., Bauer, M., Pinotsis, D., & Friston, K. J. (2016). Dynamic causal modelling of eye movements during pursuit: confirming precision-encoding in V1 using MEG. Neuroimage, 132, 175-189.

Auksztulewicz, R., Barascud, N., Cooray, G., Nobre, A. C., Chait, M., & Friston, K. (2017). The cumulative effects of predictability on synaptic gain in the auditory processing stream. Journal of Neuroscience, 37(28), 6751-6760.

Brown, H. R., & Friston, K. J. (2013). The functional anatomy of attention: a DCM study. Frontiers in human neuroscience, 7, 784.
